# The karyotype of *Nothoscordum arenarium* Herter (Gilliesioideae, Alliaceae): A populational and cytomolecular analysis

**DOI:** 10.1590/S1415-47572009005000016

**Published:** 2009-01-23

**Authors:** Luiz G. R. Souza, Orfeo Crosa, Helga Winge, Marcelo Guerra

**Affiliations:** 1 Laboratório de Citogenética Vegetal, Departamento de Botânica, Centro de Ciências Biológicas, Universidade Federal de Pernambuco, Recife, PEBrazil; 2 Laboratory of Genetics, Department of Plant Biology, Faculty of Agronomy, University of the Republic, MontevideoUruguay; 3 Departamento de Genética, Universidade Federal do Rio Grande do Sul, Porto Alegre, RSBrazil

**Keywords:** CMA/DAPI staining, 5S and 45S rDNA, heterochromatin, *Nothoscordum*, Robertsonian translocation

## Abstract

The genus *Nothoscordum* Kunth comprises approximately 20 species native to South America. Karyologically, the genus is remarkable for its large chromosomes and Robertsonian translocations. Variation in chromosome number has been recorded in a few polyploid species and it is unknown among diploids. This study presents the chromosome number and morphology of 53 individuals of seven populations of *N. arenarium* Herter (2*n* = 10). In addition, karyotype analyses after C-banding, staining with CMA and DAPI, and *in situ* hybridization with 5S and 45S rDNA probes were performed in six individuals from one population. All individuals exhibited 2*n* = 10 (6M + 4A), except for one tetraploid (2*n* = 20, 12M + 8A) and one triploid (2*n* = 15, 9M + 6A) plant. C-banding revealed the presence of CMA^+^ /DAPI ^-^ heterochromatin in the short arm and in the proximal region of the long arm of all acrocentric chromosomes. The 45S rDNA sites co-localized with the CMA ^+^ regions of the acrocentrics short arms, while the 5S rDNA probe only hybridized with the subterminal region of a pair of metacentric chromosomes. A change in the pattern of CMA bands and rDNA sites was observed in only one individual bearing a reciprocal translocation involving the long arm of a metacentric and the long arm of an acrocentric chromosome. These data suggest that, despite isolated cases of polyploidy and translocation, the karyotype of *N. arenarium* is very stable and the karyotypic instability described for other species may be associated with their polyploid condition.

## Introduction

The genus *Nothoscordum* Kunth comprises about 20 species native to South America that are mainly found in the extra-tropical regions, with *N. bivalve* (L.) Britton being the only species to occur in North America (Guaglianone, 1972). In Brazil, the genus has been recorded predominantly in the South, except for *N. gracile* (Ailton) Stearn, a common weed in the South and Southeast, and *N. pulchellum* Kunth, found in the Northeast (Nassar and Aguiar, 1978; Guerra and Felix, 2000). Guaglianone (1972) divided the genus into two sections based on reproductive and vegetative morphological characters: *Nothoscordum* and *Inodorum*. The section *Nothoscordum* is the largest one and includes *N. bivalve* and *N. pulchellum*, whereas *Inodorum* is represented by *N. gracile* [often referred to as *N. inodorum* (Ailton) Nicholson or *N. fragrans* (Ventenant) Kunth (see Stearn, 1986)], *N. arenarium* Herter and a few other species. Guaglianone (1972) stated that *N. arenarium* Herter presents transitional characters between the two sections, but she did not provide any support for this statement.

Cytologically, the *Nothoscordum* species are characterized by large chromosomes, some larger than 20 μm (Pizzolongo, 1963; Guerra and Felix, 2000), which are metacentrics (M) or acrocentrics (A). The short arms of acrocentric chromosomes often bear the nucleolus organizer region (NOR) and show a secondary constriction and sometimes a small satellite (Kurita and Kuroki, 1963; Sato *et al.*, 1979, 1980, 1982; Sato and Yoshioka, 1984; Palomino *et al.*, 1992; Guerra and Felix, 2000).

The bimodal karyotype of the *Nothoscordum* species, exhibiting metacentric chromosomes with a length equivalent to the sum of two acrocentrics, transformed the genus into a classical example of karyotypical evolution by centric fusions and fissions (Jones, 1998). The indication that Robertsonian translocations could play a major role in the evolution of the genus was first suggested by Levan and Emsweller (1938), based on the meiotic analysis of a species with 2*n* = 19 in which a trivalent composed of a long metacentric and two acrocentrics was observed. The occurrence of Robertsonian translocations in the genus is also indicated by the number of chromosome arms, which is 16 or multiples of this number (Crosa, 1972). Nuñez *et al.* (1972) and Crosa (1972) reported the existence of diploid species with 2*n* = 8 (8M) and 2*n* = 10 (6M + 4A), tetraploids with 2*n* = 16 (16M), 2*n* = 18 (14M + 4A), 2*n* = 19 (13M + 6A) and 2*n* = 20 (12M + 8A), and hexaploids with 2*n* = 26 (22M + 4A) and 2*n* = 30 (18M + 12A). The two base numbers deduced from these chromosome numbers, *x* = 4 and *x* = 5, are represented in species of the section *Nothoscordum*, whereas only species with *x* = 5 have been found in the *Inodorum* section.

The most extensively analyzed *Nothoscordum* species is *N. gracile*, characterized by 2*n* = 19 (13M + 6A) and heterochromatic bands on the short arms of the six acrocentric chromosomes and on the long arms of five acrocentrics (Kurita and Kuroki, 1963). Small subterminal bands have also been reported in the long arm of a metacentric pair (Sato *et al.*, 1979, 1980, 1982; Sato and Yoshioka, 1984; Canelada and Fernandez, 1985) and Canelada and Fernandez (1985) observed additional centromeric bands in all chromosomes.

Heterochromatin distribution was also described in *N.pulchellum* (2*n* = 10: 6M + 4A). In this species, C-banding revealed heterochromatin only in the short arms of an acrocentric pair, coinciding with the bright bands observed after staining with the fluorochrome chromomycin A_3_ (CMA^+^ bands) and with the 45S rDNA sites. The 5S rDNA sites were located in a metacentric pair not associated with heterochromatin (Guerra and Felix, 2000). The only other karyotypic information available for *Nothoscordum* species is the terminal localization of telomeric DNA in *N. striatum* (Jacq.) Kunth [= *N. bivalve* (L.) Britton] (Sykorová *et al.*, 2006). A more extensive analysis of the numerical and structural variation in natural populations of *Nothoscordum* species has not been performed.

The cytogenetic analyses of tetraploid species of the *Inodorum* section revealed a large karyotypic variability. Crosa (1972) observed 2*n* = 18 and 2*n* = 19 in different natural populations of *N. gracile*. Nuñez *et al.* (1974) reported 2*n* = 19 as the most frequent chromosome number in species of the *Inodorum* complex , but different clones of a plant with 2*n* = 19 displayed 2*n* = 18 or 20. 2*n* = 18 and 2*n* = 19 were also observed in *N. bivalve* of the *Nothoscordum* section (Palomino *et al.*, 1992). Nevertheless, little is known about the karyotypic variability in natural populations of diploid species of this genus.

*Nothoscordum arenarium* is a diploid species with 2*n* = 10, 6M + 4A (Crosa, 1972), distributed along the river banks of Uruguay, Argentina and Southern Brazil (see Guaglianone, 1972). In the present study, the karyotypic variability of this species was evaluated through the analysis of the chromosome number and morphology of 53 individuals from seven populations, including chromosome banding techniques and fluorescent *insitu* hybridization (FISH) with 5S and 45S rDNA probes in six individuals.

**Figure 1:**
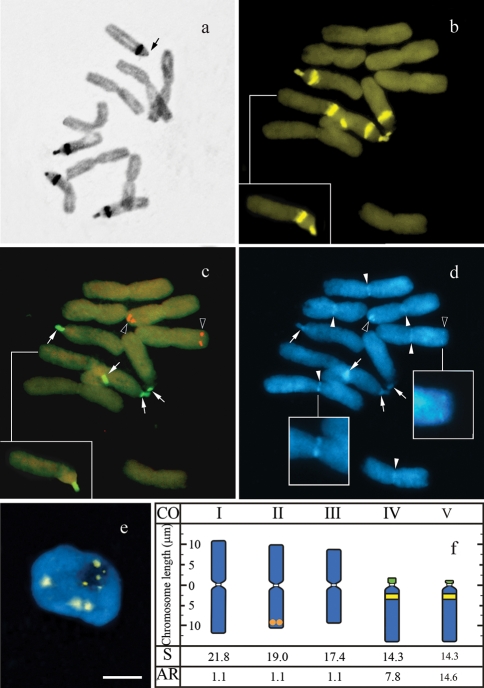
Distribution of heterochromatin and rDNA sites in the chromosomes of *Nothoscordum arenarium* metaphases after: (a) C-banding (arrow points to the smallest band); (b,d) CMA staining; (b) sequential *in situ* hybridization with 5S (red) and 45S (green) rDNA probes (merged images in c), counterstained with DAPI (d). Inserts in (b) and (c) show a digitally separated chromosome. Observe the bright DAPI bands at the centromeres in (d) (full arrowheads and higher magnification in insert) and at the 5S and 45S rDNA sites (empty arrowheads and arrows, respectively). (e) CMA/DAPI stained interphase nucleus with small chromocentres associated with the nucleolus. (f) Idiogram showing the CMA^+^ bands (yellow), 5S (red) and 45S (green) rDNA sites, and centromeric heterochromatin (white). CO, chromosome ordering; S, chromosome size; AR, arm ratio. The bar represents 10 μm.

**Figure 2:**
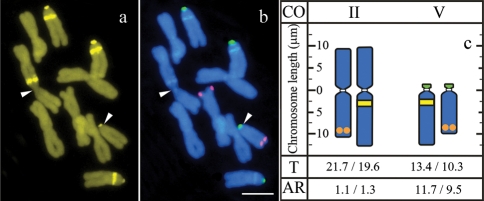
Metaphase with a reciprocal translocation after: (a) CMA staining and (b) *in situ* hybridization with 5S (red) and 45S (green) rDNA probes; arrowheads indicate the probable breakpoints and the bar represents 10 μm. (c) Idiogram as in Figure 1.

## Material and Methods

Fifty-three plants of *N. arenarium* collected at two different periods were analyzed: 47 plants from Brazil and Uruguay collected in the years 1988-1989 (sample A) and six plants from San José, Uruguay, collected in 2006 (sample B). The collection sites and number of examined individuals are shown in Table 1. The vouchers were deposited in the Herbarium of the Faculdad de Agronomía, Universidad de la República, Montevideo, Uruguay (MUFA).

Root tips obtained from bulbs were pretreated with 0.05% colchicine during 24 h at 12 °C and fixed in ethanol:acetic acid (3:1; v/v) for two to 24 h at room temperature and stored at -20 °C.

Chromosome preparations from sample A were submitted to conventional staining. In this case, the root tips were hydrolyzed in HCl 1N at 60 °C for 10 min, washed in distilled water, macerated in a drop of lactopropionic orcein and analyzed.

### Chromosome banding

Banding techniques and FISH were performed sequentially in six individuals from sample B. Fixed root tips were washed in distilled water and digested in a 2% (w/v) celullase (Onozuka) - 20% (v/v) pectinase (Sigma) solution at 37 °C for 90 min. The meristem was macerated in a drop of 45% acetic acid and the coverslip was later removed in liquid nitrogen.

C-banding was performed according to Schwarzacher *et al.* (1980). After two days aging, the preparations were hydrolyzed in 45% acetic acid for 10 min at 60 °C, denatured in a saturated solution of barium hydroxide for 10 min at room temperature, renatured in 2x SSC for 80 min at 60 °C and stained with 2% Giemsa for 30 s. For double staining with the fluorochromes CMA and DAPI, the slides were aged for three days, stained with 10 μL of CMA 0.5 mg/mL for 30 min and then stained with 10 μL of DAPI 2 μg/mL for 60 min. The slides were mounted in glycerol:McIlvaine buffer pH 7.0 (1:1) and aged for three days before analysis in an epifluorescence Leica DMLB microscope (based on Carvalho *et al.*, 2005). The images were captured with a Cohu CCD video camera using the Leica QFISH software, and later edited in Adobe Photoshop version 10.0.

### Fluorescent *in situ* hybridization (FISH)

In order to localize the rDNA sites, a 500 bp 5S rDNA clone (D2) of *Lotusjaponicus*, labeled with Cy3-dUTP (Amersham), and a 6.5 kb 18S-5.8S-25S clone (R2) of *Arabidopsisthaliana*, labeled with Spectrum Green-dUTP (Vysis), were used as probes (see Cabral *et al.*, 2006). Both labelings were performed by nick translation. FISH was performed as described by Jiang *et al.* (1995) with small modifications. The hybridization mix contained formamide 50% (v/v), dextran sulphate 10% (w/v), 2x SSC and 5 ng/μL of each probe. The slides were denaturated at 75 °C for three minutes. The final stringency of hybridization was 76%. Images of the best cells were captured as previously described.

### Chromosome measurements and idiograms

The total chromosome length (S) and the chromosomes arm ratios (AR = long/short arm) of 47 individuals from sample A were estimated from drawings using a Zeiss camera lucida coupled to a Zeiss GFL microscope. The same measurements were performed in sample B, adding the size and position of the heterocromatic blocks and rDNA sites and using the Adobe Photoshop version 10.0 software.

## Results

Fifty-one of the 53 individuals of *N. arenarium* analyzed presented the standard karyotype with six metacentric and four acrocentric chromosomes (2*n* = 10, 6M + 4A). One individual was tetraploid (2*n* = 20, 12M + 8A) and one was triploid (2*n* = 15, 9M + 6A). A single plant of sample A had a small supernumerary chromosome (c. 4.0 μm) which was only observed in some cells. A similar chromosome was also observed in three cells of a single individual from sample B. No primary constriction was observed in these chromosomes and they may represent B chromosomes or recurrent acentric fragments.

The chromosome pairs were classified from I through V according to the decreasing size of their short arms. The six metacentrics varied in size from 20.6 to 16.5 μm with a small variation in their arm ratios (Figure 1f). The two acrocentric pairs could be distinguished in most cells by the size of their short arms, which was slightly larger in pair IV than in pair V. In some cells two of the acrocentrics displayed a secondary constriction, sometimes with a small satellite.

C-banding revealed the equilocal presence of heterochromatin throughout the short arm and in the proximal region of the long arm of the acrocentric pairs (Figure 1a). After CMA and DAPI staining, all C-bands were CMA^+^/DAPI^-^ (Figure 1b). In the interphase nuclei, small CMA^+^ blocks associated with the nucleolus were observed, whereas larger CMA^+^ blocks were usually found away from it. These bright blocks apparently corresponded, respectively, to the short arm and to the proximal band of the acrocentric chromosomes (Figure 1e).

*In situ* hybridization with the 45S rDNA probe revealed four signals which co-localized with the CMA^+^ bands of the short arms of the acrocentrics, while the 5S rDNA probe hybridized with a small site in the subterminal region of the second largest metacentric pair (Figure 1c). Although no centromeric heterochromatin was observed after C-banding or CMA/DAPI staining, after FISH some cells exhibited DAPI^+^ blocks in the centromeric region of all metacentrics and more rarely in the acrocentrics (full arrowheads in Figure 1d). After FISH, DAPI^+^ blocks which co-localized with each one of the small 5S rDNA sites and sometimes also with the 45S rDNA sites (respectively, empty arrowheads and arrows in Figure 1d) were also observed. The idiogram in Figure 1f summarizes these results.

In one of the individuals from sample B one homologue of pair II presented an unusually large long arm and one of the acrocentric chromosomes had a long arm smaller than usual. Analysis of the CMA^+^ bands and rDNA sites showed that the short arm of the abnormal acrocentric was apparently unchanged and contained the 45S rDNAr site, but the long arm did not present the proximal CMA^+^ band and had a subterminal 5S rDNA site. On the other hand, the long arm of the abnormal metacentric had an interstitial CMA^+^ band and did not have the subterminal 5S rDNA site (Figure 2a and b). These data suggest that the two long arms were reciprocally translocated and the breakpoints were in the centromere or in the proximal region between the centromere and the interstitial CMA^+^ band. Measurements of the chromosome arms length in five cells of this individual indicated that the total chromosome length was not significantly affected (Figure 2c).

## Discussion

The chromosome number and morphology of *N. arenarium* observed herein was identical to that previously reported (Crosa, 1972). The heterochromatin distribution in this species, in the short arm and proximal region of the long arm of the two acrocentric pairs, was similar to the one observed in most acrocentric chromosomes of *N. gracile* (Kurita and Kuroki, 1963; Sato *et al.*, 1979, 1980, 1982; Sato and Yoshioka, 1984). On the other hand, in *N. pulchellum* (of the *Nothoscordum* section) the acrocentrics did not present bands on the long arms (Guerra and Felix, 2000), a further indication of the greater phylogenetic similarity between *N. arenarium* and *N. gracile*, both of the *Inodorum* section.

The DAPI^+^ centromeric bands observed in *N. arenarium* after FISH may correspond to a type of heterochromatin not especially rich in GC or AT which has also been reported in some other species after chromosome denaturation or after C-banding (see, *e.g.*, Besendorfer *et al.*, 2002). In *N. gracile*, centromeric C-bands were reported in all chromosomes by Sato *et al.* (1982) and Canelada and Fernandez (1985), but not by other authors, as Kurita and Kuroki (1963) and Sato *et al.* (1980). Similarly, subtelomeric C bands were observed in *Allium cepa* following the more usual protocols, while centromeric C bands were only detected under unusual technical conditions (Fiskesjö, 1974; Cortés and Escalza, 1986).

The location of the 5S rDNA in the long arms of a pair of metacentrics in *N. arenarium* was similar to that observed in *N. pulchellum* (Guerra and Felix, 2000), although slightly more terminal. The 5S rDNA site did not coincide with the C bands or CMA^+^ staining in neither species. However, in *N. arenarium,* some cells presented a small DAPI^+^ block which co-localized with the 5S rDNA site after chromosome denaturation by formamide during FISH. In the tetraploid *N. gracile* a small block of heterochromatin was also observed in two metacentric pairs in a position similar to that of the 5S rDNA site in *N. arenarium* (Sato *et al.*, 1979; Sato and Yoshioka, 1984; Canelada and Fernandez, 1985). This chromosome band has cytochemical properties different from the ones of the proximal heterochromatin of the long arm of the acrocentrics (Sato *et al.*, 1979; Sato and Yoshioka, 1984). The co-localization of 5S rDNA sites with heterochromatin is not always observed in angiosperms (Carvalho *et al.*, 2005; Fregonezi *et al.*, 2006). Its detection may depend on the technique used, the size and structural characteristics of the rDNA site (Cabral *et al.*, 2006). On the other hand, the co-localization of the C bands or the CMA^+^ bands with 45S rDNA sites is common to almost all plants (Carvalho *et al.*, 2005; Fregonezi *et al.*, 2006; Cabral *et al.*, 2006), including *N. pulchellum* (Guerra and Felix, 2000).

The occurrence of translocations in the genus *Nothoscordum* was suggested based on the comparison of the chromosomes morphology, with apparent fusion of acrocentrics or fission of metacentrics (Nuñez *et al.*, 1974; Jones, 1998; Guerra, 2008). Such translocations would have been responsible for both the variation of the basic numbers of the genus, *x* = 4 (4M) and *x* = 5 (3M + 2A), and for the formation of the tetraploid cytotypes 2*n* = 18 (14M + 4A) and 2*n* = 19 (13M + 6A). In *N. arenarium* it was demonstrated that reciprocal translocation did also occur between metacentric and acrocentric chromosomes. It is possible that the role of Robertsonian translocations in chromosome evolution of plants have been underestimated due to the lack of chromosome arm markers, such as the CMA bands and rDNA sites of *N. arenarium*. Chromosome painting in species of Brassicaceae (Lysak *et al.*, 2006), mammals (Ferguson-Smith and Trifonov, 2007) and other organisms suggest that translocations played a crucial role in karyotypic evolution of these groups.

Except for one individual with a reciprocal translocation, two polyploids and two plants with a chromosome fragment or a B chromosome, this work indicates a low intra- and interpopulational karyotype variability in *N. arenarium*, probably without any repercussion on the evolution of these populations. In another sample of eight individuals from San José (Uruguay), Crosa (1972) also observed a stable karyotype with 2*n* = 10, 6M + 4A. Guaglianone (1972) reported the occurrence of five bivalents in one plant of *N. arenarium* collected at Concordia (Argentina). Likewise, the chromosome numbers observed in a few populations of other diploid species of the genus also revealed stable karyotypes (Crosa, 1972). On the contrary, tetraploid species of the *Inodorum* section and the tetraploid *N. bivalve* of the *Nothoscordum* section exhibited extensive variability in chromosome numbers. These data suggest that the karyotype instability previously reported for this genus may be related to the polyploid condition (Crosa, 1972, Nuñez *et al.*, 1972; Palomino *et al.*, 1992). *N. pulchellum*, the only other diploid species of *Nothoscordum* which had natural populations analyzed including banding patterns and the localization of the rDNA sites, did not show any numerical or structural chromosome polymorphisms either (Guerra and Felix, 2000).

## Figures and Tables

**Table 1:** Provenance, number of individuals, chromosome number, and karyotype variations of the samples of *Nothoscordum arenarium* analyzed.

Provenance^a^	Number of individuals	2*n*	Karyotype variations^b^
Brazil: Munic. Alegrete	8	10	-
Uruguay: Dept. Colonia	3	10	Triploidy (2*n* = 15)
Uruguay: Dept. Rio Negro	3	10	Tetraploidy (2*n* = 20)
Brazil: Munic. Rosário do Sul	15	10	-
Uruguay: Dept. San José	6	10	Reciprocal translocation (2*n* = 10) B chromosome or fragment (2*n* = 10 + 1)
Brazil: Munic. Santana do Livramento	10	10	B chromosome or fragment (2*n* = 10 + 1)
Uruguay: Dept. Tacuarembó	8	10	-

^a^Munic. = municipality; Dept. = department. ^b^A single individual per sample.
